# Cross-Cultural Adaptation, Reliability, and Validity of the Greek Version of the Subjective Patient Outcome for Return to Sports (SPORTS) Score Questionnaire in Athletes with Various Shoulder Pathologies †

**DOI:** 10.3390/healthcare14091219

**Published:** 2026-05-01

**Authors:** Sotiria Vrouva, Eftychia Andreou, Georgios Krekoukias, Eleftherios Paraskevopoulos, Konstantinos Chanopoulos, George A. Koumantakis

**Affiliations:** 1 Department of Physical Therapy, 401 Army General Hospital of Athens, 11525 Athens, Greece; svrouva@uniwa.gr; 2 Laboratory of Advanced Physiotherapy, Physiotherapy Department, School of Health & Care Sciences, University of West Attica (UNIWA), 12243 Athens, Greece; mscphys25002@uniwa.gr (E.A.); gkrekoukias@uniwa.gr (G.K.); 3 School of Physical Education and Sports Science, National and Kapodistrian University of Athens, 17237 Dafne, Greece; elparaskev@phed.uoa.gr; 4 Department of Application Developments, Hellenic Military Geographical Service (HMGS), 11362 Athens, Greece; k_hanos@yahoo.com

**Keywords:** return to play, return to sport, upper extremity, injury, prevention

## Abstract

**Background:** Shoulder injury is common among athletes who engage in sports where the upper limb is actively involved. These injuries can affect an athlete’s performance and ability to return to sports at the preinjury level. This study aimed to cross-culturally adapt the Subjective Patient Outcome for Return to Sports score in Greek (SPORTS-GR) and evaluate its reliability and construct validity. **Methods:** Sixty-five Greek athletes (18–40 years) diagnosed with shoulder pathology were included. The SPORTS-GR was adapted according to standard procedures, and its construct validity was examined via associations with the Shoulder Pain and Disability Index (SPADI-GR) questionnaire, which assesses pain and disability related to shoulder pathologies. Test–retest reliability was tested by repeating the SPORTS score questionnaire administration after 6–8 days. Eligible athletes were selected from the Hellenic Supreme Council of Military Sports. **Results:** Face validity was excellent with a ceiling effect of 35.4%. The construct validity was high, with strong negative correlations between the SPORTS-GR score and the SPADI-GR total score (r = −0.91, *p* < 0.001), pain subscale score (r = −0.84, *p* < 0.001), disability subscale score (r = −0.90, *p* < 0.001), and age (r = 0.26, *p* = 0.04). Test–retest reliability was also excellent (ICC = 0.98) with no significant systematic error (SEM = 0.09). **Conclusions:** The SPORTS-GR is valid and reliable for evaluating athletes’ return to sports with shoulder pathologies.

## 1. Introduction

The shoulder is considered one of the most complex joints in the human body [1,2]. Any alteration in its structure and function, leading to modified shoulder mechanics, can result in injuries [3]. Athletes who engage in sports involving upper limb activities are particularly prone to various shoulder pathologies [1,3]. These sports include, but are not limited to, volleyball, tennis, baseball, swimming, water polo, American football, rugby, and javelin [4].

Several studies have examined the epidemiological evidence of shoulder injuries in athletes based on their sport. Hootman et al. (2007) reported that the incidence of upper extremity injuries across 15 sports over a 16-year period was 18.3% during competition and 21.4% during practice [5]. Additionally, Wright et al. (2018) reported that 30% of athletes whose sports necessitate lifting the upper extremity above shoulder height at some point in their career are at risk of injury to the shoulder girdle [6]. The highest prevalence of shoulder injuries is 58–69% among baseball athletes, 12.5–41% among cricket athletes, 7–40% among handball athletes, 14–25% among softball athletes, and 8–60% among volleyball athletes [3]. In football, shoulder problems are recorded in 15% of those playing in offensive positions, predominantly due to the severity of collisions, with only 2.1% of cases attributable to movements above the shoulder level [3].

The high prevalence of shoulder injuries in athletes can be attributed to the repetitive and intensive nature of sporting programs and activities [3,4]. Athletes involved in sports requiring overhead movements are particularly susceptible to shoulder problems because of high-speed movements and extreme shoulder joint positions [7]. Additionally, deficiencies in muscle strength and motor control often underpin injury etiology [8]. Factors such as muscle fatigue, incorrect biomechanics, and reinjury from the increased demands of the sport also contribute to the prevalence of injuries [6,8]. Common pathologies include instability, hyperextension, dislocation, shoulder muscle tendinopathies, rotator cuff tendon injuries, and impact-related injuries [9].

The primary goal for athletes undergoing postinjury sports rehabilitation is to promptly resume their sporting activities at the same level of exertion and performance as prior to the injury [8,10,11]. Although there are many questionnaires that can record shoulder pain and dysfunction [12,13], there is no questionnaire in the Greek literature that detects the ability to return to sports. The Subjective Patient Outcome for Return to Sports (SPORTS) score questionnaire is a reliable self-report tool in which an athlete selects one of five questions to determine whether they can return to sports activity at the same level as before the injury, whether with or without pain [14,15]. To date, it has been validated in populations returning to athletic activity post anterior cruciate ligament reconstruction [14] or post shoulder instability surgery [15]. Given that it can be used on any joint, it was challenging to use it in athletes with shoulder pain.

The objective of this study was to perform a cross-cultural adaptation of the Greek version of the SPORTS score to determine its reliability and to ascertain its construct validity in evaluating athletes with a variety of shoulder problems concerning their return to sport.

## 2. Materials and Methods

### 2.1. Participants

The present study was conducted between June and July 2024 in athletes of the Hellenic Supreme Council of Military Sports, Athens, Greece. The study population consisted of adult athletes between 18 and 40 years of age. The age restriction of 40 years was applied to ensure the homogeneity of the sample and to avoid including older patients with potential osteoarthritic changes in the joints of the shoulder girdle [16]. Athletes whose upper limbs were used in their sport were included in the study. Participants were required to have been diagnosed with a shoulder pathology by their doctor within the last decade, regardless of whether they had undergone surgical or conservative treatment. The study did not take into account the participant’s history of medication for shoulder injury, whether received previously or at the time of questionnaire administration. Additionally, participants were required to speak and comprehend the Greek language.

Potential participants with severe orthopedic, neurological, oncological, or cognitive problems or severe comorbidities were excluded from this study. Athletes whose shoulder injuries prevented them from returning to their sport on medical advice were also excluded. Finally, participants who could not speak or comprehend Greek were also excluded.

Eligible athletes were informed about the study and gave their written consent to participate. Questionnaires were distributed in printed form and completed by the patients themselves.

The study was approved by the Ethics Committee of the University of West Attica (protocol number 44487/31 May 2024).

### 2.2. Procedures

The cross-cultural adaptation methodology followed the process outlined in Beaton et al. (2000) [17]. Specifically, after permission was obtained from the creator of the SPORTS score, two translations from English to Greek were conducted by bilingual translators, one health professional and one without clinical experience in the health sector. The two versions were discussed and combined, and an agreed upon version of the questionnaire was created. A ‘backward translation’ of this initial Greek version into English was subsequently performed to compare it with the original English version, which was carried out by two bilingual translators who permanently resided in Greece and whose native language was English.

Then, a review process was followed, involving a committee consisting of at least one bilingual individual with excellent knowledge of Greek and English, a health professional, and the translators. This committee produced the prefinal version of the SPORTS score in Greek. This version was pilot-tested on 10 athletes with shoulder problems to assess the comprehensibility of the questions and identify any points to be resolved, according to the guidelines for the cross-cultural adaptation process of self-report measures [17]. Since no issues were identified, the questionnaire’s format was finalized.

The participants signed a consent form after being thoroughly informed about the aims of the study. They subsequently completed the demographic data section, which included questions about gender, age, marital status, education, and occupation. The participants then completed the two self-report questionnaires, the SPORTS score (Supplementary Material) and the Shoulder Pain and Disability Index (SPADI) in Greek, which were administered on two separate occasions, with the second occurring 6–8 days after the initial time of administration.

### 2.3. Subjective Patient Outcome for Return to Sports (SPORTS) Score

The SPORTS score questionnaire evaluates athletes’ readiness to resume activities postinjury [14,15,18]. It includes five response categories reflecting the athlete’s current condition. These categories are based on three concepts: (1) performing the same sport with the same or less effort, (2) performing at the same or reduced level, and (3) performing with or without pain. Scores range from 0 (unable to return) to 10 (returning to preinjury levels) [14]. Athletes who stopped their sport for non-shoulder-related reasons reported the highest score since the injury [18].

### 2.4. Shoulder Pain and Disability Index (SPADI)

The Shoulder Pain and Disability Index (SPADI) is a questionnaire used for self-assessment of shoulder pain and disability in individuals with various shoulder conditions [19]. The SPADI questionnaire includes 13 questions, with the first five evaluating pain and the remaining eight assessing dysfunction. Each question uses a numerical rating scale (NRS) ranging from 0 (no pain and no difficulty) to 10 (maximum pain and difficulty in movement requiring assistance). Patients choose the score that best represents their experience of shoulder issues in the past week. The total score ranges from 0 to 130, where 0 indicates no shoulder disability and 130 signifies maximum shoulder disability. The total score is calculated by summing the responses and converting them to a percentage [19]. The SPADI-GR is a cross-culturally adapted, valid and reliable version of the scale in Greek [12], and this version was administered to the participants of this study.

The overall SPADI score is divided into subgroups of 20 points to interpret the results more effectively. Therefore, the following categories are commonly used: 0–20, mild shoulder pain and dysfunction; 21–40, moderate shoulder pain and dysfunction; 41–60, severe shoulder pain and dysfunction; 61–80, very severe shoulder pain and dysfunction; and 81–100, extreme shoulder pain and dysfunction [19]. A change in score of 10 to 20 points indicates a significant improvement or deterioration in shoulder pain and dysfunction [19].

### 2.5. Statistical Analysis

In order to estimate the required number of patients for the establishment of any statistical relation as significant, appropriate sample size calculation and a priori power analysis were performed [20]. For that reason, we utilized the G*Power 3.1 calculator [21,22] for the correlation tests we would use for construct validity and a web-based sample size calculator for reliability studies [23]. The parameters applied in both calculators were a level of significance a = 0.05 and a statistical power of at least >80%. Furthermore, the input parameter applied for the correlation tests was the expected effect size. Although we expected a high correlation between the SPORTS score and SPADI, we used a small-to-moderate effect size of 0.35 to reduce the probability of the results being attributed to chance. Similarly, the parameters applied for estimating the intraclass correlation coefficient (ICC) reliability index were the minimum acceptable reliability of ICC:0.8, the expected reliability of ICC:0.9, number of raters/repetitions per subject of k:2 and expected dropout rate of 10%. Both calculators showed that by using 65 patients in total, we could secure 80% power for all the assessments performed.

The statistical analysis was conducted via the software package IBM SPSS Statistics 29.0.2.0 for Windows (SPSS Inc, Chicago, IL, USA). *p*-values less than 0.05 were considered statistically significant. The Kolmogorov–Smirnov test was used to assess the normal distribution, which showed that the SPORTS-GR score data exhibited a nonnormal distribution (*p* < 0.05). Exploratory analysis by subgroups was carried out using nonparametric tests (Mann–Whitney U for 2 groups, Kruskal–Wallis for 3+ groups) in order to evaluate differences in rank-ordered or non-normally distributed numerical data across independent groups.

Descriptive statistics were presented as the means and standard deviations (±SDs) for quantitative data and as frequencies and percentages (%) for qualitative data.

First, the descriptive–demographic statistics of the participants, along with their values in the SPORTS-GR and SPADI-GR questionnaires were provided, according to the type of each variable. Specifically, the maximum, minimum, and mean values of the variables, as well as the standard deviation, were examined.

### 2.6. Test–Retest Reliability

The ICC was used to quantify the test–retest reliability or stability over time of the SPORTS-GR score, indicating the extent to which consistent test results are obtained upon repeated assessments when no real change is expected [24]. The ICC_2,1_ random effects absolute agreement was calculated for the agreement between the two responses (test and retest after 6–8 days) for the SPORTS-GR. The ICC ranges from 0 (no agreement) to 1 (perfect agreement), with values above 0.75 indicating excellent reliability, values between 0.4 and 0.75 indicating fair to good reliability, and values below 0.4 indicating poor reliability [25]. In addition, Cohen’s weighted kappa was calculated, suitable for determining the agreement for ordinal variables, given the categorical nature of the SPORTS questionnaire [26]. Cohen’s kappa coefficient ranges from −1 (complete disagreement) to 1 (complete agreement).

As an indicator of absolute reliability, the standard error of the measurement (SEM) was also calculated. It is the standard deviation of errors of measurement related to test–retest, measured in the same units as the SPORTS score [27].

The data were further analyzed via Bland–Altman analysis to define the “limits of agreement”. This method relies on the mean and standard deviation of the differences between ratings of the same subject. Bland and Altman recommended that 95% of data points should fall within 2 standard deviations of the mean difference, accounting for systematic bias at ±1.96 [28].

### 2.7. Face and Content Validity

Face validity and content validity pertain to the ability of the questionnaire to conceptually encompass the range of the variable it measures. The questionnaire was submitted for evaluation to a panel of experts regarding the concept of ‘self-assessment regarding athletes’ return to their sport after a shoulder injury’. The panel evaluated the relevance of the questionnaire’s items to this concept via a five-point scale, where 1 indicated ‘not relevant’ and 5 indicated ‘completely relevant’ [29].

### 2.8. Construct Validity

Construct validity evaluates whether the measure under study accurately represents a particular construct or concept [27]. This type of validity is determined by correlating the measure with another variable that is already recognized as a valid measure of the construct. The criterion validity of the SPORTS-GR score was assessed by calculating the correlation coefficient between the SPORTS-GR score and the overall SPADI-GR score and its subscales, namely, pain and disability. Owing to the nonnormal data distribution (Kolmogorov–Smirnov test, *p* = 0.001), nonparametric tests using the Spearman correlation coefficient were conducted. As defined in the literature, correlation coefficients below 0.25 are considered weak, those between 0.25 and 0.50 are considered fair, those between 0.50 and 0.75 are considered moderate to good, and those above 0.75 are considered good to excellent [27]. High correlations between instruments with similar constructs confirm the convergent validity, whereas low correlations between instruments with different constructs confirm discriminant validity [27].

## 3. Results

### 3.1. Descriptive Statistics

**Figure 1:**
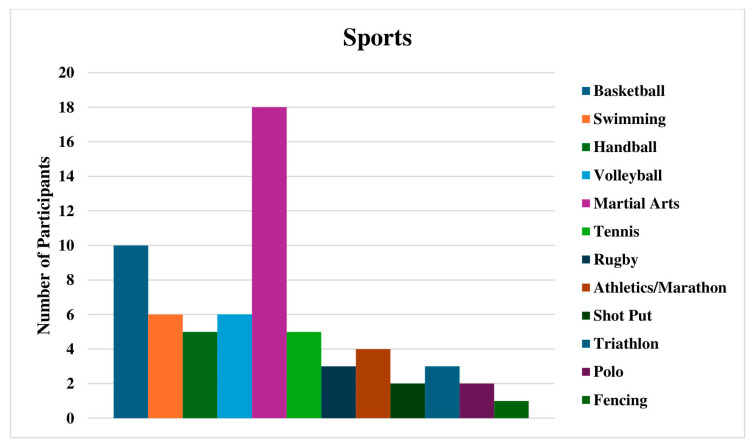
Distribution of participants in relation to their sport.

**Figure 2:**
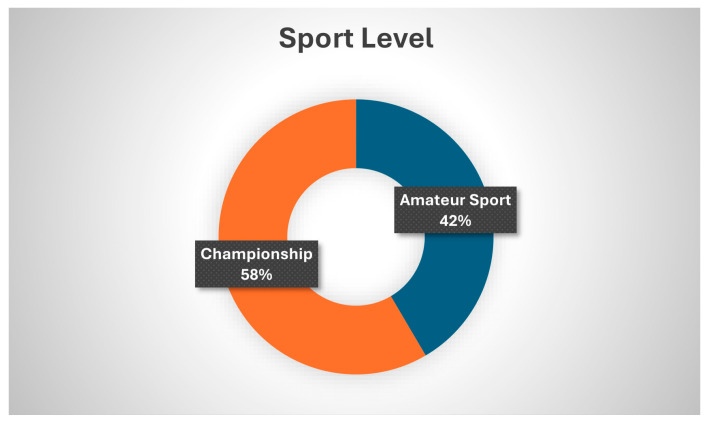
Distribution of participants by level of sport.

**Figure 3:**
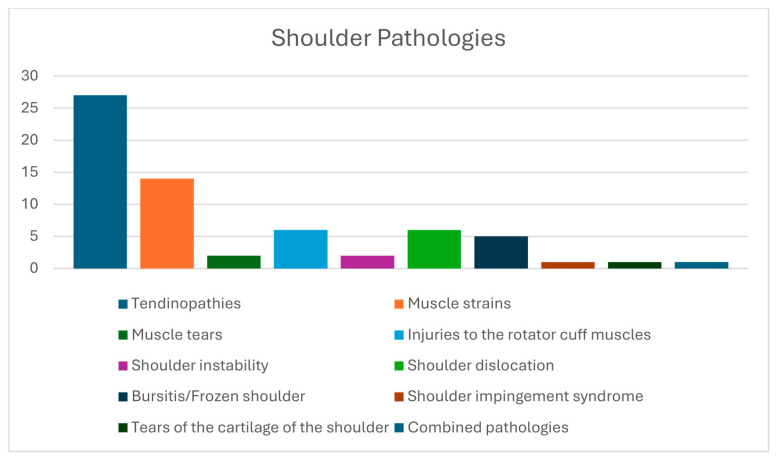
Distribution of participants in relation to their shoulder pathology.

A total of 65 patients (40 men and 25 women) with a mean age of 28.03 ± 6.14 years (range: 18–40 years) and a mean body mass index (BMI) of 22.25 ± 1.36 kg/m^2^ (range: 19–26.5 kg/m^2^) met the eligibility criteria and participated in the study (Table 1). Thirty-four participants (52.3%) reported pain or dysfunction in the left shoulder, fifty-five (86.4%) had completed their treatment, and the majority were treated conservatively (89.2%) with physiotherapy and medication. Graphs display the distribution of participants relative to their sport (Figure 1), sport level (Figure 2), and shoulder pathology (Figure 3).

**Figure 4:**
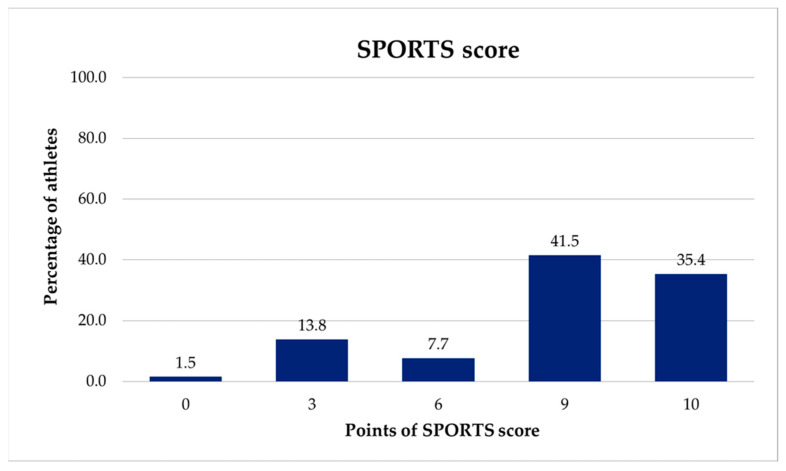
Distribution of the SPORTS-GR scores. 0, Unable to return to same sport; 3, Perform same sport but at reduced level of effort and performance compared with before the onset of impairment; 6, Perform same sport at same level of effort but reduced performance level compared with before onset of impairment; 9, Perform same sport at same level of effort and performance as before onset of impairment but with pain; 10, Perform same sport at same level of effort and performance as before onset of impairment and with no pain.

For the SPORTS-GR score, fifty patients (76.9%) reported that they were able to return to the same sport activity at the same level of effort and performance as before the onset of injury, with or without any pain; two patients reported an increase of one level and one patient reported a decrease of one level (Figure 4). On the first measurement occasion, the average score for the SPORTS-GR score was 8.15 ± 2.58 and the average total SPADI-GR score was 23.6 ± 23.99 (range: 0–79.2) (Table 1).

Furthermore, Mann–Whitney U test results did not indicate any significant differences in the SPORTS-GR score between the two categories of sport level (*p* = 0.248) and between the two different treatments received (*p* = 0.289). Similarly, a Kruskal–Wallis test showed that the distribution of SPORTS-GR was the same across categories of sport practiced (*p* = 0.289). Specifically, the mean SPORTS-GR score was 8.1 ± 2.60 for championship level and almost the same for the amateur level 8.23 ± 2.60. Athletes treated conservatively were found to have a mean of 8.24 ± 2.47 versus those treated surgically who had a mean of 7.43 ± 3.50. Finally, regarding the sport practiced, the lower average values were found for rugby and handball, 5.0 ± 4.30 and 5.0 ± 3.464, respectively.

### 3.2. Face Validity

A panel of experts assessed the Greek version of the SPORTS questionnaire to test face validity. The panel concluded that all phrases were relevant to athletes’ self-assessment of their return to sport. Each phrase received a score of 5 from all expert group members on a 5-point scale (1: ‘not relevant’ and 5: ‘absolutely relevant’). The translations closely matched the original English questionnaire.

### 3.3. Content Validity

**Figure 5:**
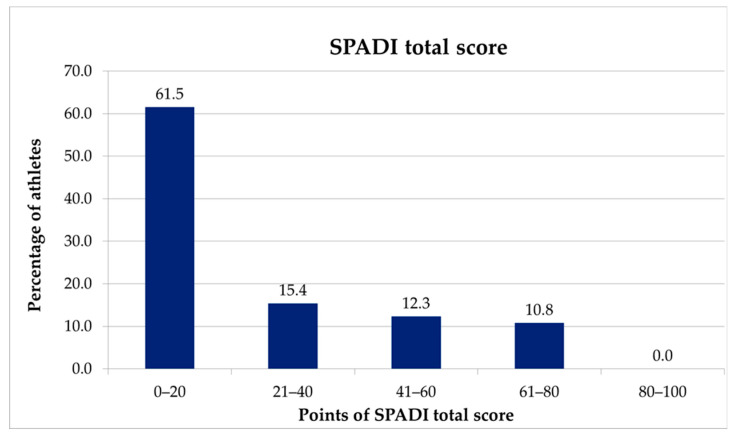
Distribution of total SPADI scores categorized into subgroups of 20 points. The highest reported scores translated to the worst conditions of the patient.

The percentage of patients reporting the highest (10) and lowest (0) possible scores on the SPORTS-GR score questionnaire was calculated to assess floor and ceiling effects, which are components of content validity. Specifically, ceiling effects occur when a significant percentage of participants achieve the highest possible score, whereas floor effects occur when a significant percentage achieve the lowest possible score, with values between 0 and 15% considered acceptable [29]. Values of 9% and 32% for ceiling and floor effects respectively were reported for the SPORTS score [14]. For the current study, these effects were displayed graphically for both questionnaires (Figure 4 and Figure 5). Given that the minimal detectable difference for the overall SPADI score is approximately 18 points, patient-reported scores were divided into subgroups of 20 points.

Twenty-three participants (35.4%) returned to their sport at the same level of effort and performance without pain, and 27 (41.5%) returned with some pain. Additionally, 14 participants (21.5%) returned to their sport, with five athletes (7.7%) maintaining the same level of effort but lower performance and nine (13.8%) experiencing lower levels of effort and performance. Only one participant was unable to return to their sport. The SPORTS-GR score exhibited an acceptable floor effect of 1.5% and a high ceiling effect of 35.4%, lower than that reported in a previous validation [15]; however, it was well above the 15% threshold. Similarly, the ceiling effect of the total SPADI-GR score recorded for the participants in this study was high (61.5%) and showed a good distribution of the outcome if it is taken into account that highest reported scores translated to the worst conditions of the patient (Figure 5).

### 3.4. Construct Validity

Spearman’s correlation coefficient between the SPORTS-GR score and the total SPADI-GR score was found to be very strong and negative (r = −0.91, *p* < 0.001), indicating that high scores of SPORTS-GR scores were associated with low SPADI-GR scores. Significant negative correlations were also observed between the SPORTS-GR score and the SPADI-GR subscales: a very strong correlation with the pain subscale (r = −0.84, *p* < 0.001) and with the disability subscale (r = −0.90, *p* <0.001). Additionally, a weak but significant positive correlation was identified between the SPORTS-GR score and age (r = 0.26, *p*-value = 0.04).

### 3.5. Test–Retest Reliability

**Figure 6:**
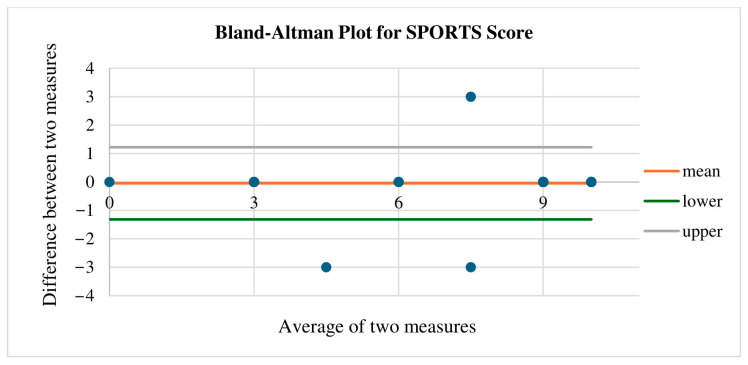
Bland–Altman plot for SPORTS-GR score. Y-axis: the difference between two paired measurements of SPORTS. Orange line: the mean difference: the mean difference found was −0.09, indicating that the SPORTS-GR scores, two weeks later on average, differed from the initial evaluation by −0.09. Gray and green lines: ±1.96 standard deviations from the mean difference. Bland and Altman recommended that 95% of data points should fall within 2 standard deviations of the mean difference. This 95% confidence level is commonly associated with z-scores of ± 1.96, which are shown on this particular chart. X axis: mean of the two measures (A + B)/2.

Test–retest reliability for the SPORTS-GR score questionnaire was found to be excellent (ICC_2,1_ = 0.98; 95% confidence interval = 0.96–0.99). In addition, the kappa measure of agreement was found to be 0.91, slightly over 0.9, which also indicates excellent reliability. The SEM indicating the error level between the first and second administrations of the SPORTS-GR score was calculated, revealing a systematic error of 0.09 points with a 95% upper limit of agreement of 1.22 points. Bland and Altman analysis revealed that almost 95% of the data points fell within two standard deviations of the mean difference (Figure 6), indicating that there was no significant systematic error. Specifically, out of 65 participants, 62 provided the same answers in both test–retest measurements, whereas three patients had different responses at the second evaluation. One patient initially scored nine points and later scored six points after two weeks. The other two patients noted an increase of 3 points at the second administration (one patient from 3 to 6 points and one from 6 to 9 points).

On the first measurement occasion, the average total score for the SPADI-GR was 23.6 ± 23.99 (range: 0–79.2), and on the second measurement occasion, it was 22.81 ± 22.87 (range: 0–81.5).

## 4. Discussion

Sporting activities carry the risk of injury, particularly for athletes involved in upper limb sports [4]. The shoulder is the most mobile and complex joint in the human body [2], and as such, injuries can significantly impact performance [1]. These injuries may compel an athlete to temporarily withdraw from sporting activities [10,11]. Therefore, assessing an athlete’s performance postinjury is crucial.

The SPORTS score questionnaire serves as an evaluation tool to assess athletes’ readiness to resume sporting activities after an injury [18]. However, it has not been adapted to the Greek language. Blonna et al. (2012) utilized it to evaluate athletes 5–10 years after anterior cruciate ligament surgery [14]. It has also been used to assess athletes following shoulder instability surgery [15] and at 6 and 12 months after anterior cruciate ligament surgery [18]. In the latter study, a modified version of the SPORTS questionnaire was used, replacing “same sport” with “any sports” [18]. The SPORTS score was determined to be valid and reliable across all the aforementioned studies.

Therefore, the objective of this study was to cross-culturally adapt the SPORTS score questionnaire, resulting in a final Greek version that would be easily comprehensible to participants, to ascertain its reliability through repeated administration to athletes with shoulder disorders, and to evaluate its construct validity by comparing it to the SPADI-GR (convergent validity) [12]. Cross-cultural adaptation of questionnaires is essential for the consistent use of tools across diverse scientific communities. This process involves translating questionnaires into local languages, ensuring comprehension across different medical disciplines within independent nations. It is an accepted method for creating questionnaires that are applicable in multiple countries, as developing new questionnaires is both costly and time-consuming. Concurrently, the adapted tool has been validated by assessing its reliability and validity [17].

The study included 65 athletes (40 men and 25 women), aged 18 to 40 years old, who were proficient in Greek. These athletes had been diagnosed with a shoulder pathology by their treating physician within the last decade, irrespective of whether they had received surgical or conservative treatment.

Additionally, the observed rates of ceiling and floor effects are considered satisfactory. Previous studies on the content validity of the SPORTS questionnaire are cited. Specifically, Blonna et al. (2012) investigated the validity and reliability of the SPORTS questionnaire among 47 athletes and reported a floor effect of 9%, which was deemed acceptable, and a ceiling effect of 32%, exceeding the acceptable threshold [14], but still lower than those reported in the Knee Injury and Osteoarthritis Outcome Score (KOOS) questionnaire, which evaluates both short- and long-term outcomes following a knee joint injury [30], and the Lysholm questionnaire, which is used for assessing symptoms and function in patients with various knee injuries [31]. In another study by Blonna et al. (2014) involving 98 athletes, an acceptable baseline rate of 8% and a ceiling rate of 46% were reported [15], which was higher than the SPORTS-GR ceiling effect (35.4%) but better than the ceiling effect noted in the Rowe score, the Oxford Shoulder Instability Score (OSIS), and the Western Ontario Shoulder Instability Index (WOSI) tools. Specifically, the Rowe score assesses shoulder joint mobility, stability, and functionality [32]. The OSIS questionnaire is used to evaluate shoulder instability in daily living activities [33]. The WOSI questionnaire measures shoulder function concerning physical symptoms, pain, sports, recreation, work, lifestyle, social functioning, and emotional well-being [13]. The high ceiling rate becomes more apparent when comparing treatments yielding positive outcomes, as statistically significant differences are identified. Additionally, in the study conducted by Bley et al. (2022) involving 90 patients, no floor effect was detected, while the ceiling effect slightly exceeded the 33% threshold at 12 months postrehabilitation [18].

Finally, a high ceiling effect, where over 35% of patients achieve the maximum score (e.g., [10/10] on the SPORTS-GR score), acts as a significant limitation in measuring true recovery, as it prevents distinguishing between patients who are fully recovered and those who are slightly better than that. While it indicates excellent clinical outcomes, it reduces the score’s ability to detect further improvements, often highlighting that the assessment criteria may be too easy for that population [34]. This means that while the outcome is positive for the patient, the tool itself loses sensitivity to differentiate between very good and excellent outcomes or else it indicates a low discriminatory power for the instrument and limits the ability to measure further improvement over time (longitudinal studies) [35]. Regarding the extent to which the athletes return to sports, exploratory analyses by subgroups showed that there were not significant differences due to the type of treatment or the sport they practiced and the level at which they played. However, this interpretation of the results should be approached with caution because of the high ceiling effect.

The construct validity of the SPORTS-GR score is considered to be high. In the existing literature, studies examining the construct validity of the SPORTS in various contexts have used Spearman’s correlation coefficient to derive results. Specifically, Blonna et al. (2012) utilized the Spearman correlation coefficient to analyze the relationships between the SPORTS score and (a) the Lysholm questionnaire score, (b) the KOOS questionnaire total score, (c) the KOOS questionnaire subscales on sport and recreation, and (d) the Short Form 36 (SF-36) scale [14]. In Blonna’s study, the SPORTS score data exhibited a nonnormal distribution (Kolmogorov–Smirnov test, *p* = 0.001) [14]. This study revealed a moderate relationship between the SPORTS score and the total KOOS (r = 0.51, *p* = 0.001) and its sport and recreation subscales (r = 0.55, *p* < 0.001). The coefficients between the SPORTS score and the Lysholm total score (r = 0.43, *p* = 0.0026) and between the SPORTS score and the SF-36 scale (r = 0.399, *p* = 0.0054) are considered fair [14].

In a subsequent study by Blonna et al. (2014), construct validity was ascertained by calculating Spearman’s correlation coefficient between the SPORTS score and (a) the WOSI questionnaire score, (b) the Rowe score, (c) the OSIS questionnaire score, and (d) the Subjective Shoulder Value (SSV) questionnaire score [15]. The SSV questionnaire is a self-completed patient questionnaire that asks patients to rate their shoulder joint compared with the ‘normal’ shoulder [36]. In this study, a moderate-to-strong correlation was observed between the SPORTS score and all the questionnaires, except for the WOSI questionnaire’s lifestyle and social functioning scale (r = 0.40, *p* = 0.005). The strongest correlation was identified between the SPORTS questionnaire and the SSV questionnaire (r = 0.60, *p* < 0.001) and between the SPORTS questionnaire and the “sport, leisure and work” parameter of the WOSI questionnaire (r = 0.57, *p* < 0.001) [15].

Finally, in their study, Bley et al. (2022) [18] used the Spearman correlation coefficient between the SPORTS score and the Tegner, Lysholm, KOOS-sport/recreation, and Marx questionnaires at both time points, at 6 and 12 months after anterior cruciate ligament reconstruction, to determine the construct validity. Fair construct validity was found at 6 months between the same and any sport of the SPORTS questionnaire and the KOOS-sport/recreation questionnaires (r = 0.47 for both subscales), Lysholm (r = 0.35 and 0.33), Marx Activity Rating Scale (r = 0.35 and 0.33), and Tegner (r = 0.31 and 0.32) scores (*p* < 0.001). At 12 months after rehabilitation, Spearman’s correlation coefficients were also considered fair between the same and any sport of the SPORTS score and the Tegner questionnaire (r = 0.45 and 0.43), the Lysholm (r = 0.39 for both), the KOOS-sport/recreation (r = 0.38 for both subscales), and the Marx Activity Rating Scale (r = 0.31 for both) scores (*p* < 0.001) [18]. The Tegner Activity Scale is a one-item questionnaire used to assess activity level [31], whereas the Marx Activity Rating Scale is a 5-point Likert scale used to assess running, directional changes, slowing down, and rotation [37].

As mentioned, the instrument showed good construct validity. The SPORTS-GR score, in fact, showed a strong negative correlation (r = −0.91) with SPADI, a reliable and valid tool for shoulder pain and disability. It is worth noting that the magnitude of the correlation coefficient between SPORTS and SPADI is considerably larger than that of the other shoulder assessment tools used in the aforementioned studies, in which it did not exceed 0.6. This can be interpreted as a strong correlation between return to sport and the subjective feeling of pain and disability of the shoulder. As expected, better correlation was found between the SPORTS score and the disability subscale of SPADI (r = −0.91) than that with the pain subscale (r = −0.84). The patients who scored 0–20 in the SPADI, compared with those who scored over 20, had a better chance of returning to their full sporting activity without pain.

The test–retest reliability of the SPORTS questionnaire was examined via the ICC_2,1_, the SEM, and the Bland and Altman ‘limits of agreement’ with the re-administration of the questionnaire occurring after a two-week interval. The results demonstrated excellent reliability, as no significant deviations in participants’ responses were observed between the two administrations. Previous studies examining the reliability of repeated measures of the SPORTS score reported similar outcomes. Specifically, Blonna et al. (2012) observed excellent reliability (ΙCC = 0.967; 95% confidence interval, 0.94–0.98) with a systematic error of 0 points (95% maximum agreement = 1.8) [14]. Additionally, another study by Blonna et al. (2014) reported excellent reliability after 2–3 weeks (ICC = 0.95; 95% confidence interval, 0.93–0.97), with a systematic error of 0.3 points and a 95% upper limit of agreement of 2.3 points [15]. However, Bley et al. (2022) reported moderate-to-good test–retest reliability at 1 year for both the SPORTS score related to returning to the same sport (ICC = 0.58; 95% confidence interval, 0.4–0.72) and the modified SPORTS score related to the ability to return to any sport (ICC = 0.60; 95% confidence interval, 0.43–0.73) [18].

A number of limitations in this research should be acknowledged, although they did not impede the completion of the study. Because of the high ceiling effect, the interpretation of the results should be approached with caution. Furthermore, studies evaluating the return to sport of athletes with shoulder disorders via SPORTS score are lacking. Moreover, the sample size (n = 65) was limited, although it was sufficient for data collection and extraction of valid and reliable results. Additionally, participants in the study did not undergo a clinical examination by the study’s investigators. Additionally, the investigators did not communicate with the study participants’ treating physician regarding their shoulder pathology. The only assessment performed was when the questionnaires were completed. Future research should involve a larger sample size to further explore this topic, with the ultimate goal of retesting reliability and validity for specific subcategories of shoulder impairment.

## 5. Conclusions

The Greek version of the SPORTS score is valid and reliable for evaluating athletes’ return to sports with shoulder pathologies. Future research should focus on athletes with other joint pathologies.

## Figures and Tables

**Table 1:** Patient demographics.

	All Participants (N = 65)
Age ^a^, years	28.03 ± 6.14
Body mass index ^a^, kgr/m^2^	22.25 ± 1.36
Sex ^b^ (male/female)	40 (61.5)/25 (38.5)
Injured shoulder ^b^ (left/right)	31 (47.7)/34 (52.3)
Treatment ^b^ (surgery/conservative)	7 (10.8)/58 (89.2)
SPORTS ^a^	8.15 ± 2.58
SPADI Total ^a^	23.6 ± 23.99
SPADI Pain ^a^	26.58 ± 25.41
SPADI Disability ^a^	20.76 ± 23.30

^a^ Values are expressed as the mean ± SD; ^b^ values are expressed as number of patients (%).

## Data Availability

The datasets generated and/or analyzed during the current study are not publicly available due to the applicable data protection law in Greece (Law 4624/2019) but are available from the corresponding author on reasonable request.

## Supplementary Materials

The following supporting information can be downloaded at https://www.mdpi.com/article/10.3390/healthcare14091219/s1, Table S1: Final Greek version of the SPORTS score questionnaire.

## Abbreviations

The following abbreviations are used in this manuscript:

ICC	Intraclass Correlation Coefficient
SEM	Standard Error of the Measurement
SPADI	Shoulder Pain and Disability Index
SPORTS	Subjective Patient Outcome for Return to Sports
