# Supplementary material for: Cross-Cultural Adaptation, Reliability, and Validity of the Greek Version of the Subjective Patient Outcome for Return to Sports (SPORTS) Score Questionnaire in Athletes with Various Shoulder Pathologies
Source: Healthcare (Basel). 2026 May 1;14(9):1219. doi: 10.3390/healthcare14091219 (PMC13164102; doi:10.3390/healthcare14091219)
Supplement: Supplementary file 1 [file healthcare-14-01219-s001.zip › healthcare-4179055-supplementary.pdf]

Final Greek version of the SPORTS Score questionnaire

| SPORTS Score – GR [a] | | |
|---|---|---|
| **Κατηγορία** | **Ορισμός** | **Σχετική τιμή** |
| Προσπάθεια χωρίς περιορισμό<br>Επίδοση χωρίς περιορισμό<br>Καθόλου πόνος | Εκτέλεση του ίδιου αθλήματος στο ίδιο επίπεδο προσπάθειας και επιδόσεων όπως με εκείνο πριν από την έναρξη της βλάβης και χωρίς καθόλου πόνο | 10 |
| Προσπάθεια χωρίς περιορισμό<br>Επίδοση χωρίς περιορισμό<br>Κάποιος/ μερικός πόνος | Εκτέλεση του ίδιου αθλήματος στο ίδιο επίπεδο προσπάθειας και επιδόσεων όπως με εκείνο πριν από την έναρξη της βλάβης αλλά με πόνο | 9 |
| Χωρίς περιορισμό προσπάθεια<br>Περιορισμένη επίδοση | Εκτέλεση του ίδιου αθλήματος στο ίδιο επίπεδο προσπάθειας αλλά με μειωμένο επίπεδο επιδόσεων σε σύγκριση με εκείνο πριν από την έναρξη της βλάβης | 6 |
| Περιορισμένη προσπάθεια<br>Περιορισμένη επίδοση | Εκτέλεση του ίδιου αθλήματος αλλά με μειωμένο επίπεδο προσπάθειας και επιδόσεων σε σύγκριση με εκείνο πριν από την έναρξη της βλάβης | 3 |
| Αναπηρία | Αδυναμία επιστροφής στο ίδιο άθλημα | 0 |
| [a] SPORTS, Subjective Patient Outcome for Return to Sports Score-Greek Version | | |

Original SPORTS Score questionnaire

| SPORTS Score[a] | | |
|---|---|---|
| Category | Definition | Relative Value |
| Unlimited effort<br>Unlimited performance<br>No pain | Perform same sport at same level of effort and performance as before onset of impairment and with no pain | 10 |
| Unlimited effort<br>Unlimited performance<br>Some pain | Perform same sport at same level of effort and performance as before onset of impairment but with pain | 9 |
| Unlimited effort<br>Limited performance | Perform same sport at same level of effort but reduced performance level compared with before onset of impairment | 6 |
| Limited effort<br>Limited performance | Perform same sport but at reduced level of effort and performance compared with before onset of impairment | 3 |
| Disabled | Unable to return to same sport | 0 |

[a]SPORTS, Subjective Patient Outcome for Return to Sports.
